# Minimally invasive treatment for breast cancer metastasis to the esophagus

**DOI:** 10.1186/1753-6561-9-S1-A50

**Published:** 2015-01-14

**Authors:** MA Wilson, ME O’Donnell, DE Jaroszewski, KL Harold

**Affiliations:** 1 Royal College of Surgeons in Ireland, 123 St. Stephen’s Green, Dublin 2, Ireland; 2 Division of Vascular and Endovascular Surgery, Mayo Clinic, Arizona, USA; 3 Division of Thoracic Surgery, Mayo Clinic, Arizona, USA; 4 Division of General Surgery, Mayo Clinic, Arizona, USA

## Background

Breast cancer metastasis to the esophagus is a rare phenomenon affecting 0.03% of patients with advanced breast cancer.This remains a diagnostic challenge due to frequent asymptomatic presentation.

## Methods

We present the successful minimally invasive surgical (MIE) treatment of an isolated metastatic breast lesion to the esophagus.

## Results

A sixty-two year-old female presented in May 2009 with an eighteen-month history of dysphagia due to a chronic benign esophageal structure, presumed secondary to previous radiotherapy treatment for breast cancer. She complained of occasional heartburn, indigestion and cough and described a 60lbs weight loss due to tolerance of a liquid only diet. She had a fifteen-year smoking history. She had been undergoing monthly esophageal dilatations over the previous six-months. Multiple previous esophageal biopsies were benign. Clinical assessment was unremarkable. Endoscopic ultrasound demonstrated a tight fibrotic stricture at 26cm.Additional biopsies were again negative for malignancy. She was referred for MIE surgical resection. After creation of the pneumoperitoneum and insertion of four trocars, the short gastric vessels were divided followed by mobilisation of the gastric fundus with preservation of the gastroepiploic artery. High mediastinal dissection was performed to mobilize the esophagus followed by a chemical pyloromyotomy. A mini-right posterior-lateral thoracotomy identified a small caliber esophagus which was dissected free of right bronchial adhesions. The esophagus was subsequently divided proximally and distally followed by a stapled anastomosis. Histopathological analysis confirmed an invasive adenocarcinoma consistent with a breast primary. She remains well four-years post-surgery. Unfortunately, in advanced cases, therapeutic interventional strategies tend to target symptomatic palliation rather than curative resection. Conventional open esophagectomy involves a myriad of incisions depending on the tumour site. These incisions create significant patient morbidity.MIE surgery has evolved to minimise patient morbidity compared to conventional open techniques. Shorter operative times without the need to re-position the patient is cost-effective, whilst preservation of the latisimus dorsi muscle may reduce post-operative pain and improve overall quality of life (QOL) post operatively. The four laparoscopic port sites provide adequate abdominal exposure whilst the mini-thoracotomy facilitates esophageal mobilisation and division. Higher physical function index scores have been reported twenty-four weeks following MIE surgery compared to conventional open surgeries.

## Conclusions

Despite a lack of randomized trials comparing MIE with the conventional open techniques, current evidence suggests that less invasive interventions improve peri-operative patient experience with improved QOL after surgery.

